# Production and Screening of High Yield Avermectin B1b Mutant of *Streptomyces avermitilis* 41445 Through Mutagenesis

**DOI:** 10.5812/jjm.8626

**Published:** 2014-02-01

**Authors:** Samia Siddique, Quratulain Syed, Ahmad Adnan, Fahim Ashraf Qureshi

**Affiliations:** 1 Department of Chemistry, Government College University Lahore, Lahore, Pakistan; 2 Food and Biotechnology Research Center, PCSIR Laboratories Complex, Ferozpur Lahore, Lahore, Pakistan; 3 Office of Research, Innovation and Commercialization, Comsats Institute of Information and Technology, Islamabad, Pakistan

**Keywords:** Avermectin B1b, Mutagenesis, *Streptomyces avermitilis*, Submerged Fermentation, Hereditary Stability

## Abstract

**Background:**

Secondary metabolite production from wild strains is very low for economical purpose therefore certain strain improvement strategies are required to achieve hundred times greater yield of metabolites. Most important strain improvement techniques include physical and chemical mutagenesis. Broad spectrum mutagenesis through UV irradiation is the most important and convenient physical method.

**Objectives:**

The present study was conducted for enhanced production of avermectin B1b from *Streptomyces avermitilis* 41445 by mutagenesis using ultraviolet (UV) radiation, ethidium bromide (EB), and ethyl methanesulfonate (EMS) as mutagens.

**Materials and Methods:**

*S. avermitilis* DSM 41445 maintained on yeast extract malt extract glucose medium (YMG) was used as inoculum for SM2 fermentation medium. Spores of *S. avermitilis* DSM 41445 were exposed to UV radiation for physical broad spectrum mutagenesis and to EMS and EB for chemical mutagenesis. For each mutagen, the lethality rate and mutation rate were calculated along with positive mutation rate.

**Results:**

Avermectin B1b-hyper-producing mutant, produced using these three different methods, was selected according to the HPLC results. The mutant obtained after 45 minutes of UV radiation to the spores of *S. avermitilis* 41445, was found to be the best mutant for the enhanced production of avermectin B1b component (254.14 mg/L). Other avermectin B1b-hyper-producing mutants, were obtained from EMS (1 µL/mL) and EB (30 µL/mL) treatments, and yielded 202.63 mg/L and 199.30 mg/L of B1b, respectively.

**Conclusions:**

The hereditary stability analysis of the UV mentioning 45 minutes revealed the UV exposure time for mutants and 3 represented the colony taken from the plate irradiated for 45 minutes mutant showed that the production of avermectin B1b remained constant and no reverse mutation occurred after 15 generations.

## 1. Background

*Streptomyces avermitilis* is an aerobic, Gram positive and mesophilic *Actinomycete*, forming extensively branched substrate mycelium and aerial hyphae. The hyphae are differentiated into long, compact spiral chains which become more open as the culture ages, and are specialized for the production of the avermectin complex (1). Avermectins are secondary metabolites having anthelmintic and insecticidal properties and have extensively been used in agricultural and animal health cases. They have been produced from *S. avermitilis* by fermentation (1). Strain improvement strategies and better production conditions are very important to enhance the yield of secondary metabolites in any fermentation process (2). The concentration on secondary metabolites produced from wild strains is very low due to the complicated economical procedure (3).

Hundred times greater yields of metabolites can be achieved through suitable strain improvement techniques (4). Mutagenesis is the most reliable and widely-used tool for strain improvement (5). Induced mutations using UV rays, X-rays, γ-rays, laser, neutron, and chemophoresis are in practice for organism breeding (6). Methyl methane sulfonate (MMS), hydroxyl amine (HA), and N-methyl-N-nitro-N-nitrosoguanidine (MNNG) are the physical methods to induce mutations (7). MNNG is highly specific in producing GC-AT transition mutations and this limits its usefulness as a mutagen (8).

Chemical modification of nucleotides is supposed to be induced using alkylating agents such as Ethylmethane Sulfonate (EMS). It results in mispairing and base changes in the nucleotide sequence (9). EMS mutagenesis is useful for producing breeding lines (10). EMS produces C-T changes resulting in C/G and T/A substitutions (11). 7-ethylguanidine hydrolysis results in G/C to C/G or G/C to T/A transversions while 3-ethyl adenine pairing errors cause A/T to G/C transitions (12). Treatment with ethidium bromide (EB) usually results in bald mutants leading to no sporulation (13).

The most important and convenient physical method to obtain broad spectrum mutations is UV radiation. It is safe to use UV light as a mutagen, compared to chemical mutagenesis (14). Improved secondary metabolite production from industrial microbe strains has been obtained by random mutagenesis and fermentation screening (15). Most of the *Streptomyces* members are genetically unstable, and morphologically stable mutants are needed for strain improvement strategies (16, 17). 

## 2. Objectives 

**Figure 1.**
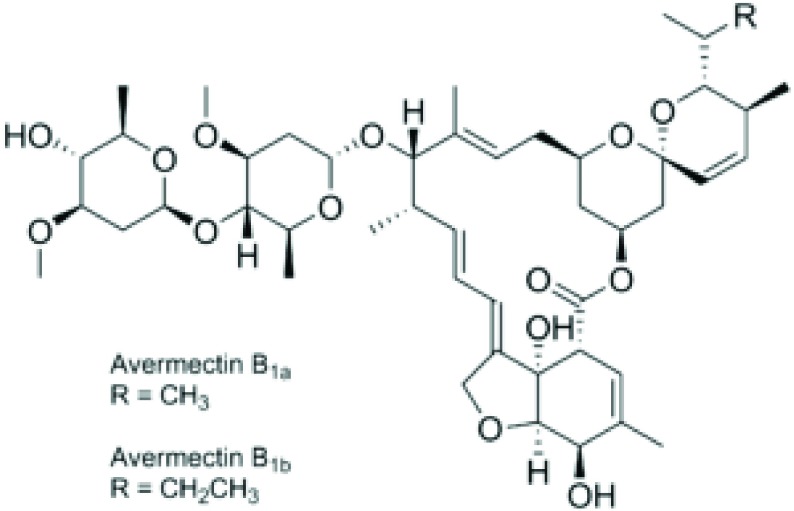
Avermectin Chemical Structure

The present study was conducted for production and screening of avermectin B1b (Figure 1) hyper-producing mutant strain of *S. avermitilis* 41445 by means of physical (UV radiation) and chemical mutagenesis (ethyl methane sulfonate and ethidium bromide). The main objective of the study was to enhance the production of avermectin B1b through mutagenesis.

## 3. Materials and Methods

### 3.1. Microorganism and Maintenance of Culture

*S. avermitilis* DSM 41445 provided by Deutsche Sammlung von Mikroorganismen and Zellkulturen (DSMZ) GmbH was used throughout the study. *S. avermitilis* DSM 41445 was maintained on medium 65 as specified by DSMZ. The medium 65 (Yeast extract malt extract glucose medium) (Merck, Germany) consisted of glucose 4.0 g, yeast extract 4.0 g, malt extract 10.0 g, and CaCO_3_ 2.0 g (g/L in distilled water) (18). The medium was adjusted to pH 6.5 before sterilization. After sterilizing at 121°C for 15 minutes, the medium pH was adjusted at 7.0 by adding CaCO_3_ (20). The medium was then inoculated with *S. avermitilis* 41445 and incubated at 28^o^C in a water bath shaker at 150 X g until it was converted to a brownish liquid. Nutrient agar (Merck, Germany) slants were used for the culture to streak, followed by incubation at 28^o^C for 24 hours to be stored. All the mutant microbial cultures were maintained on nutrient agar slants (2.8%w/v 2.8 g nutrient agar dissolved in 100ml of distilled water). The incubation temperature for the culture growth was 28°C, because the *S. avermitilis* culture grows well at 28°C and 37°C (2).

### 3.2. Experimental Protocol for Microbial Mutation

#### 3.2.1. UV Mutagenesis

The *S.* *avermitilis* DSM 41445 spores were exposed to UV rays at a distance of 10 cm from the UV lamp (DESAGA, Sarstedt-Gruppe, MinUVIS UV Lamp) (λ = 320nm) for 5, 10, 15, 20, 25, 30, 35, 40, 45, 50, 55, and 60 minutes. All these exposures were performed in a dark room to avoid any photoreaction in the production of mutants. The spores were then spread in plates containing nutrient agar. The plates were incubated at 28^o^C for 24 hours The method employed for UV mutagenesis was described earlier by Khattab in 2012 (19) and used in the present study with little modifications. The lethality rate, mutation rate, and positive mutation rate were calculated using the below equations (20):

Lethality rate = No. of colonies after UV treatment / No. of colonies without UV treatment (T/U) × 100%

Mutation rate = total colony formation unit of the mutant strain / No. of colonies after UV treatment (M/T) × 100%

Positive mutation rate = CFU of the mutants with avermectin B1b production more than that of the parent *S.avermitilis* DSM 41445 / total colony formation unit of the mutant strain (P/M) × 100%

Here, U = No. of colonies without UV treatment

T = No. of colonies after UV treatment

M = total colony formation unit of the mutant strain

P = CFU of the mutants with avermectin B1b production more than that of the parent *S.avermitilis* DSM 41445.

The plates with highest lethality rate obtained after UV exposure were selected and all the colonies from those plates were incubated in nutrient agar slants for 24 hours at 28°C. The selected mutant colonies were further screened through avermectin production ability to determine the positive mutation rate (20).

#### 3.2.2. Chemical Mutagenesis

For chemical mutagenesis, a 12-hour-old culture of *S.* *avermitilis* 41445 in nutrient broth was used to make the serial dilutions up to 10^-7^ in sterilized normal saline. Various concentrations (10 µL/mL, 20 µL/mL, 30 µL/mL, and 40 µL/mL) of EB were used for the mutation of *S.* *avermitilis* 41445 per mL of 10^-7^ dilution. Effect of each concentration was studied at different time intervals (10, 20, 30, 40, 50 and 60 minutes).

Similarly in case of EMS, 1 µL of EMS was used for 1 mL of dilution. Then, 0.3 mL of the above dilution was taken and poured in the Petri plates each of which containing nutrient agar. The plates were incubated at 28°C for 24 hours. Thereafter, the percentage of survival rate in each plate was calculated using the equation mentioned below, and mutants with the lowest survival rates were selected from the plates (21).

S = (Ni - Nd /Ni) × 100

Where, S = survival rate

Ni = Initial CFU

Nd = Colony formation unit (CFU) after mutation

#### 3.2.3. Seed Medium

A loopful of the 24-hour-old culture of *S.* *avermitilis* 41445 and all the selected produced mutants were transferred discretely into sterilized seed media. All seed media were placed at 31°C in a water bath shaker (Eyela, Japan) for 24 hours at 150 X g. The seed medium with pH 7.0 ± 2 contained glucose 4.0 g, yeast extract 4.0 g, malt extract 10.0 g, and CaCO_3_ 2.0 g (g/L in distilled water) (22).

### 3.3. Production of Avermectin B1b

Production of avermectin B1b from *S.* *avermitilis* 41445 and all the produced selected mutants were studied independently in growth medium named Synthetic medium 2 (SM2). Each production medium was inoculated with 5 mL (10% v/v) of inoculum medium separately. After transferring the seed medium, each growth medium was incubated at 31°C in water bath shaker for 10 days at 150 X g. Composition of the growth medium was soluble corn starch 50.0 g, KCl 0.1 g, NaCl 0.5 g, Yeast extract 2.0 g, MgSO_4_7H_2_O 0.1 g, CaCO_3_ 0.8 g and α-amylase 0.1 g (g/L). pH of the medium was adjusted at 7.2 ± 0.2. All the experiments were performed in shake flasks containing 50 mL of fermentation medium separately, using the method described earlier (23).

### 3.4. Extraction of Avermectin B1b

The fermentation broth from each fermentation flask was centrifuged at 4°C for 20 minutes at 8000 X g (H-1500FR, Japan) for the extraction of avermectin. Avermectin being an intracellular molecule has to be extracted from the cell biomass, and for this purpose, cell biomass was taken and the supernatant was discarded. The cell biomass in the form of pellet was mixed with an appropriate amount of methanol in a pestle and motor and was crushed hardly to be completely dissolved. The mixture was then centrifuged for separation of the cell biomass and the supernatant was collected for the analysis of avermectin by HPLC.

### 3.5. HPLC Analysis of Avermectin B1b

For quantitative determination of avermectins produced from *S.* *avermitilis* 41445 and all the mutant strains, reverse phase HPLC was employed. About 20 µL of each extracted sample was applied into HPLC (LC-2080 Shimadzu) where C18 column (SMA C-18) and detector (UV variable wavelength detector STD-M20A Shimadzu) were used for separation of components, and individual components were eluted by methanol: acetonitrile (98 : 2 v/v) at a flow rate of 0.5 mL/min with a UV absorbance at 246 nm (24).

## 4. Results

**Figure 2.**
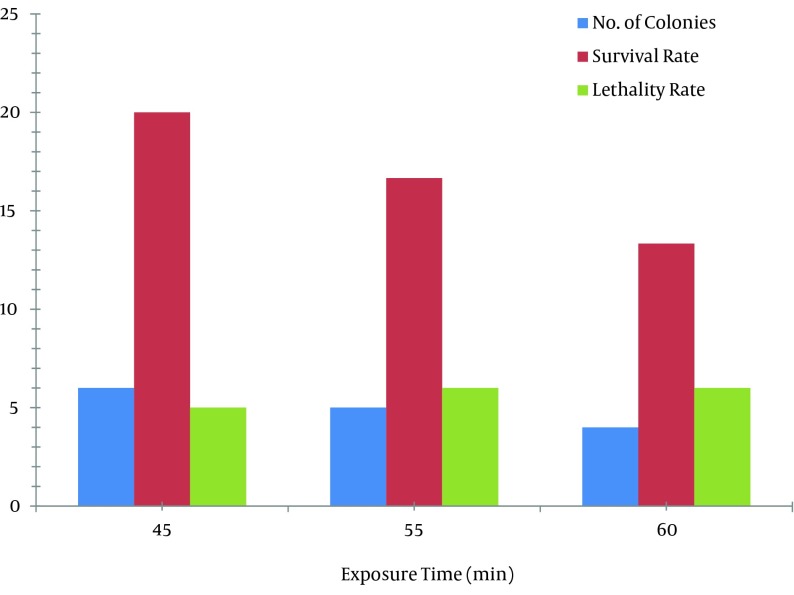
Effects of Different UV Radiation Times of on the Survival and Lethality Rates

**Figure 3.**
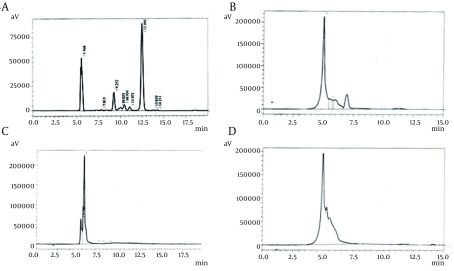
HPLC Chromatogram (A) Standard solution of Abamectin, (B) B1b produced from the EB 30 strain (minutes) 2, (C) B1b produced from the UV 45 strain (minutes) 3, (D) B1b produced from the EMS 50 strain (minutes) 1

UV exposure for 45, 55, and 60 minutes resulted in 20%, 16.66%, and 13.33% survival rates, respectively, as shown in Figure 2. The lethality rate increased from 0.05 to 0.075 with exposure time elevation from 45 to 60 minutes. In our findings, death rate of 80 - 88% was noted in the case of UV radiation. Number of mutant colonies decreased with increase of exposure time. Maximum increase in avermectin B1b production was observed in mutants obtained at exposure time of 45 and 55 minutes. From the HPLC results, it was found that the avermectin B1b-hyper-producing mutant strain obtained from UV radiation was UV 45 (3), which resulted in 14.9496 ± 0.2 folds increase (254.1443 mg/L) in the B1b component as compared to that of the *S. avermitilis* 41445 (17 mg/L) after 10 days of fermentation, as shown in Figure 3 C. Based on the avermectin B1b production, the estimated mutation rate (RM) was 92.22% and the positive mutation rate (RP) was 8.42% in the present study.

In case of EMS, 1 µL/mL concentration of EMS was used for mutation. At the exposure times of 10, 30, and 50 minutes, the survival rate was decreased from 0.769% to 0.384% and the lethality rate was increased from 1.300% to 2.604%. The mutant obtained at exposure time of 50 minutes, yielded 11 times more production of avermectin B1b (202.63 mg/L) than the parent strain (17 mg/L) as shown in Figure 3 D. 

**Figure 4.**
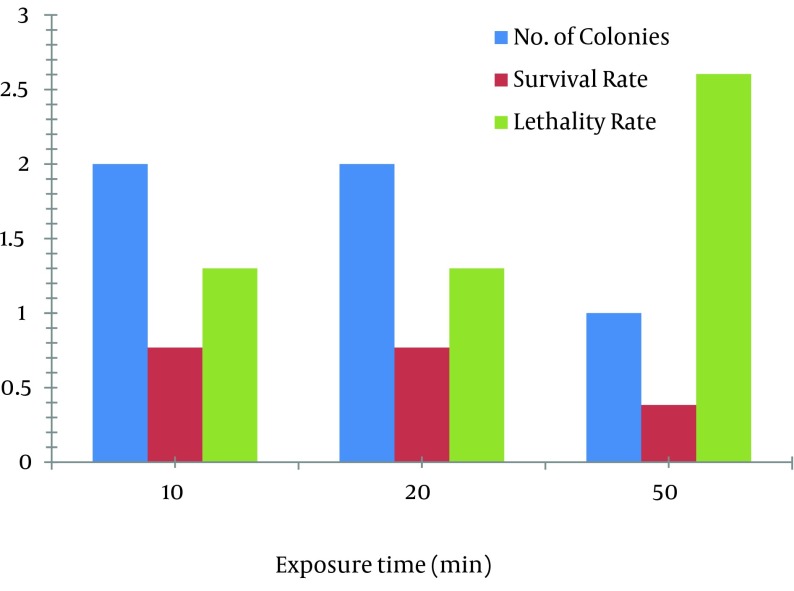
Effects of Different EMS (1 µL/mL) Treatment Times on the Survival and Lethality Rates

Two mutants obtained at the exposure time of 20 minutes yielded 10.5 times more avermectin B1b production (179.93 mg/L and 192.06 mg/L) as compared to the wild strain of *S.* *avermitilis* 41445. Production of B1b component of avermectin by the two mutants obtained at the exposure time of 10 minutes was 90.01 mg/L. Effects of EMS on the survival and lethality rates at various times are shown in Figure 4. 

## 5. Discussion

The strain improvement strategies especially mutagenesis and screening of hyper-producing mutants are very important in the production of secondary metabolites during the fermentation process (25). The genome sequences of *S.* *avermitilis* species have been known, but the mechanisms of genes involved in the avermectin production are still not clearly understood (26). UV radiation and chemical mutagenesis have been employed to obtain the avermectin hyper-producing mutants (27). 

In the present study, physical and chemical mutageneses have been employed to mutate *S.* *avermitilis* 41445 to obtain the avermectin B1b hyper-producing mutant. Physical mutagenesis was done using UV radiation. In a previous study, it was reported that to have powerful mutations and effective screening of mutants, lethality rate should be very high (20). 

It is reported that the death rate of 70 - 95% has been optimized for enhanced secondary metabolite production (28). Kelner reported an exponential survival curve with variable UV exposures to spores of *S. flaneolus* except at the beginning, where the killing rate was less than the later portions of the curve. In their study, mutation frequency arose steadily with exposure time, with no evidence of failure rate of increase at high doses (29). It is reported that a decrease in the survival rate of *S. venezuelae* from 100% to 8% occurred as a result of UV mutagenesis when the time of UV exposure was increased from 0 to25 minutes (21). In another study, survival rates of 8% and 5% were observed at UV exposure of 100 and 120 seconds, respectively, showing a gradual reduction in the survival rate with increasing UV exposure on *S. fradiae* NRRL-2702. The mutants at these exposure times resulted in enhanced tylosin production, compared to the wild type strains (30). 

It is reported that genes, expression of which are globally regulated, are required for the production of avermectin (31). Results of the present study indicated that the enhanced production of avermectin B1b might be due to some changes in the genetic code of *S.* *avermitilis* 41445 as a result of UV radiation. It is reported that chromosomes of *S.* *avermitilis* become genetically unstable with UV radiation; thus, mutations in chromosomes occur when UV light falls on the spores (24).

Production of avermectin is directly related to the large number of genes. Over-mutagenesis should therefore be avoided while screening for hyper-producing mutant strain. Optimum dose of mutagen is required in order to get positive mutation. According to the Poisson model of mutagenesis, 37% of the survival rate would be unaffected for enhanced metabolic production (7). Chemical mutagenesis is time and concentration dependent. In the present study, spores of *S.* *avermitilis* 41445 were treated with four different concentrations i.e. 10, 20, 30 and 40 µL/mL of EB for time intervals ranging from 10 to 60 minutes. 

Avermectin B1b-hyper-producing mutant was obtained from the spores treated with 30 µL/mL concentration of EB in 30 minutes of exposure (199 mg/L of B1b) as shown in Figures 3 A, 3 B 3 A. At lower concentrations of EB (10 and 20 µL/mL), the obtained mutants showed almost similar activities (17 mg/L B1b) as was shown by parent strains. In a study conducted by Naveena et al. it was mentioned that by increasing the concentration of chemical mutagens, the survival capacity of the mutants get adversely affected (21). In the present study, it is also observed that at a higher EB concentration (40 µL/mL), obtained mutants did not show any production and it resulted in the loss of activity. EB exposure at 50 minutes with 30 µL/mL concentration resulted in the survival rate of 1.33 % with lethality rate of 0.751% shown in Table 1. 

**Table 1.** Effects of Different Concentrations of EB on the Survival and Lethality Rates

Conc. of EB	Exposure Time	Colonies, No.	Survival Rate, %	Lethality Rate
**10**				
	0	150	100	0.01
	10	140	93.33	0.010
	20	140	93.33	0.010
	30	130	86.66	0.011
	40	120	80	0.012
	50	120	80	0.012
	60	100	66.66	0.015
**20**				
	0	150	100	0.01
	10	100	66.66	0.015
	20	90	60	0.016
	30	90	60	0.016
	40	80	53.33	0.018
	50	80	53.33	0.018
	60	70	46.66	0.021
**30**				
	0	150	100	0.01
	10	5	3.33	0.300
	20	10	6.66	0.150
	30	4	2.66	0.375
	40	10	6.66	0.150
	50	2	1.33	0.751
	60	8	5.33	0.187
**40**				
	0	150	100	0.01
	10	10	6.66	0.150
	20	10	6.66	0.150
	30	8	5.33	0.187
	40	8	5.33	0.187
	50	6	4.00	0.25
	60	6	4.00	0.25

In the previous study, it was also reported that treatment of *Streptomyces* with EB resulted in the bald mutants, and the mutants were not able to produce the earthy odors (32). This reveals that production of secondary metabolites and the structural differentiation are closely linked.

In an earlier research it was testified that prolonged incubation of EMS resulted in DNA damage, causing cells death. A suitable selection of exposure times is therefore mandatory to accomplish a good and fruitful chemical mutagenesis (21). In one of the studies conducted previously, MMS was used as a mutagen for the enhanced production of avermectin from *S.* *avermitilis*. This mutagen produces a mutant with 4 times more AVM B1 production than the parent strain (1). EMS Mutations followed the error-prone pathway and directly affected the mispairing of the alkylating bases (33).

**Figure 5.**
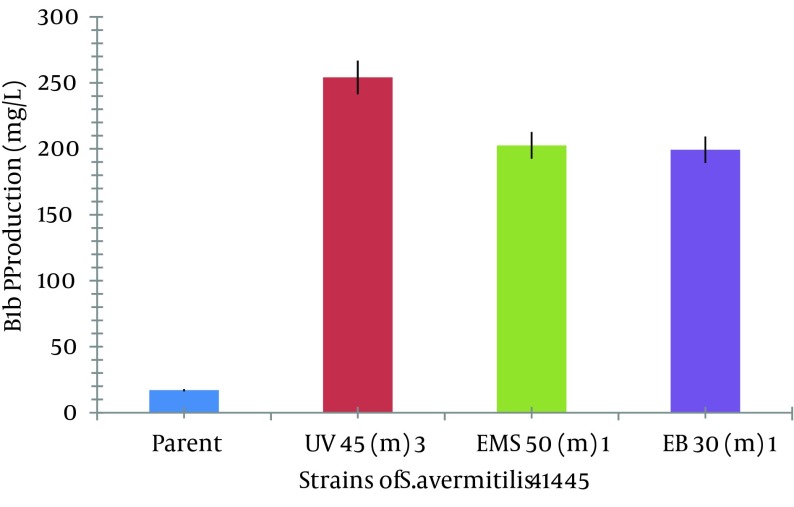
Comparative Analysis of Production of Avermectin B1b by Different Mutants With the Parent Original Strain, *S. avermitilis* 41445

Mutants obtained from different sources usually present variations in the avermectin production (33). Our results also showed that mutants produced using different mutagens had different B1b production rates. In each case, the production was enhanced when compared with the B1b production from the parent strain, *S.* *avermitilis* 41445, as shown in Figure 5. 

**Table 2.** Comparative Production of avermectin B1b From Different Mutated Strains

Serial No.	Type of Mutagen	Exposure Time, mmin	Colonies, No.	Number of Colonies Producing avermectin B1b	Concentration of B1b Produced, mg/L
**1**	UV				
		45	6	2	UV (45) 1=43.51; UV (45) 3=254.14
		55	5	3	UV (55) 1=72.43; UV (55) 2=200.27; UV (55) 3=64.11
		60	4	4	UV (60) 1=64.70; UV (60) 2=31.46; UV (60) 3=49.34; UV (60) 4=106.35
**2**	EMS				
		20	2	2	EMS (10) 1 = 90.01; EMS (10) 2 = 90.01
		20	2	2	EMS (20)1 = 179.93; EMS (20)2 = 192.06
		50	1	1	EMS (50)1 = 202.63
**3**	EB				
		10	5	2	EB (10)1 = 70.68; EB (10)2 = 119.48
		30	4	3	EB (30)1 = 138.43; EB (30)2 = 199.30; EB (30)3 = 63.17
		50	2	2	EB (50)1 = 52.03; EB (50)2 = 84.96

The mutant strain of *S.* *avermitilis* 41445, numbered 3, obtained by UV radiation after 45 minutes of exposure, showed the maximum avermectin B1b production and was selected as the avermectin B1b-hyper-producing strain. The mutant was named as *S.* *avermitilis* 41445 UV 45 (m) 3 and will be used in the further studies.

In a study conducted earlier, high avermectin producers and avermectin aglycon mutants were obtained from *S.* *avermitilis* using NTG and UV radiations as mutagens (35). In the present study, the genetic stability of avermectin B1b-hyper-producing mutant of *S.* *avermitilis* 41445 UV 45 (m) 3 was observed by multiple streaking of the mutant on nutrient agar slants and then analyzing the production of desired avermectin B1b component by HPLC, as shown in Table 3. For isolation of the stable mutants, a strategy has been developed to select a NTG-produced mutant for hyper production of spiramycin (36). About 56-time-enhanced avermectin production has been obtained from *S.* *avermitilis* ATCC 31267 with mutagenesis (2).

**Table 3.** Analysis of Heriditary Stability of UV 45(m) 3 Strain

Generation	Avermectin B1b Production, mg/L
**1**	254.14
**3**	260.18
**5**	230.56
**7**	245.36
**9**	251.67
**11**	258.61
**13**	235.81
**15**	240.98

The criterion of random selection of mutant was adopted for the screening of mutants. EB concentration of 30 µL/mL with the exposure time of 30 minutes was found to be the most effective for yielding mutants of *S.* *avermitilis* 41445 with enhanced avermectin B1b production. For EMS mutagenesis, a concentration of 1 µL/ML and exposure time of 50 minutes was found to be appropriate for enhanced production of avermectin B1b. In case of physical mutagenesis by UV radiation, the suitable exposure time to produce the B1b-hyper-producing mutant of *S.* *avermitilis* 41445 was 45 minutes. Both physical and chemical mutageneses proved to be efficient in producing hyper-producing mutant strains; however, the best mutagen found in the present study was UV radiation, yielding 14 times more avermectin B1b than the parent strain (17 mg/L).

Therefore, the mutant produced by UV radiation at exposure time of 45 minutes was selected and will be used for further studies.

## References

[1] Kim SB (2002). Streptomyces avermitilis sp. nov., nom. rev., a taxonomic home for the avermectin-producing streptomycetes.. Int J Systematic Evolution Microbiol..

[2] Burg RW, Miller BM, Baker EE, Birnbaum J, Currie SA, Hartman R (1979). Avermectins, New Family of Potent Anthelmintic Agents: Producing Organism and Fermentation.. Antimicrob Agents Chemother..

[3] Demain AL, Aharonowitz Y, Martin JF, Vining LC (1983). Metabolic control of secondary biosynthetic pathways..

[4] Demain AL, Adrio JL (2008). Contributions of microorganisms to industrial biology.. Mol Biotechnol..

[5] Volff J‐N, Altenbuchner J (1998). Genetic instability of the Streptomyces chromosome.. Mol Microbiol..

[6] Yu Chen, Zhixin Lin, Zuyao Zou, Feng Zhang, Duo Liu, Xianghuai Liu (1998). High yield antibiotic producing mutants of Streptomyces erythreus induced by low energy ion implantation.. Nucl Instrum Meth B..

[7] Kornillowicz-Kowalska T, Bohacz J (2011). Biodegradation of keratin waste: Theory and practical aspects.. Waste Manag..

[8] Baltz RH, Stonesifer J Mutagenesis in Streptomyces. Rivista di Biologia/Biology Forum.

[9] Greene Elizabeth A, Codomo Christine A, Taylor Nicholas E, Henikoff Jorja G, Till Bradley J, Reynolds Steven H (2003). Spectrum of chemically induced mutations from a large-scale reverse-genetic screen in Arabidopsis.. Genetics..

[10] Lee SY, Cheong JI, Kim TS (2003). Production of doubled haploids through anther culture of M1 rice plants derived from mutagenized fertilized egg cells.. Plant Cell Rep..

[11] Kovalchuk I, Kovalchuk O, Hohn B (2000). Genome-wide variation of the somatic mutation frequency in transgenic plants.. EMBO J..

[12] Krieg David R (1963). Ethyl methanesulfonate-induced reversion of bacteriophage T4rII mutants.. Genetics..

[13] Redshaw PA, McCann PA, Sankaran L, Pogell BM (1976). Control of differentiation in streptomycetes: involvement of extrachromosomal deoxyribonucleic acid and glucose repression in aerial mycelia development.. J Bacteriol..

[14] Miller JH (1983). Mutational specificity in bacteria.. Annu Rev Genet..

[15] Parekh S, Vinci VA, Strobel RJ (2000). Improvement of microbial strains and fermentation processes.. Appl Microbiol Biotechnol..

[16] Adrio JL, Demain AL (2006). Genetic improvement of processes yielding microbial products.. FEMS Microbiol Rev..

[17] Schrempf H (1982). Plasmid loss and changes within the chromosomal DNA of Streptomyces reticuli.. J Bacteriol..

[18] Gao H, Liu M, Zhou X, Liu J, Zhuo Y, Gou Z (2010). Identification of avermectin-high-producing strains by high-throughput screening methods.. Appl Microbiol Biotechnol..

[19] Khattab AA, Mohamed SAA (2012). Mutation induction and protoplast fusion of Streptomyces spp. for enhanced alkaline protease production.. J Appl Sci Res..

[20] Wang LY, Huang ZL, Li G, Zhao HX, Xing XH, Sun WT (2010). Novel mutation breeding method for Streptomyces avermitilis using an atmospheric pressure glow discharge plasma.. J Appl Microbiol..

[21] Naveena B, Gopinath KP, Sakthiselvan P, Partha N (2012). Enhanced production of thrombinase by Streptomyces venezuelae: kinetic studies on growth and enzyme production of mutant strain.. Bioresour Technol..

[22] Gao H, Liu M, Liu J, Dai H, Zhou X, Liu X (2009). Medium optimization for the production of avermectin B1a by Streptomyces avermitilis 14-12A using response surface methodology.. Bioresour Technol..

[23] Siddique S, Syed Q, Adnan A, Nadeem M, Irfan M, Ashraf Qureshi F (2013). Production of Avermectin B1b From Streptomyces avermitilis 41445 by Batch Submerged Fermentation.. Jundishapur J Microbiol..

[24] Chen Zhi, Wen Jia, Song Yuan, Wen Ying, Li JiLun (2007). Enhancement and selective production of avermectin B by recombinants of Streptomyces avermitilis via intraspecific protoplast fusion.. Chin Sci Bull..

[25] Zhang J, Greasham R (1999). Chemically defined media for commercial fermentations.. Appl Microbiol Biotechnol..

[26] Yong JH, Byeon WH (2005). Alternative production of avermectin components in Streptomyces avermitilis by gene replacement.. J Microbiol..

[27] You DW, Chen J, Chu J, Zhuang YP, Zhang SL, Luo JL (2005). Screening of high yield avermectins producing strain by complex mutation.. Chin J Antibiot..

[28] Simpson IN, Caten CE (1979). Induced quantitative variation for penicillin titre in clonal populations of Aspergillus nidulans.. J Gen Microbiol..

[29] Kelner A (1948). Mutation in Streptomyces flaveolus Induced by X-rays and Ultraviolet Light.. J Bacteriol..

[30] Khaliq S, Akhtar K, Afzal Ghauri M, Iqbal R, Mukhtar Khalid A, Muddassar M (2009). Change in colony morphology and kinetics of tylosin production after UV and gamma irradiation mutagenesis of Streptomyces fradiae NRRL-2702.. Microbiol Res..

[31] Chater Keith F, Bibb Mervyn J (2008). Regulation of bacterial antibiotic production.. Biotechnol Set..

[32] Redshaw PA, McCann PA, Pentella MA, Pogell BM (1979). Simultaneous loss of multiple differentiated functions in aerial mycelium-negative isolates of streptomycetes.. J Bacteriol..

[33] Stonesifer J, Baltz RH (1985). Mutagenic DNA repair in Streptomyces.. Proc Natl Acad Sci U S A..

[34] Ikeda H, Takada Y, Pang CH, Tanaka H, Omura S (1993). Transposon mutagenesis by Tn4560 and applications with avermectin-producing Streptomyces avermitilis.. J Bacteriol..

[35] Ikeda H, Kotaki H, Omura S (1987). Genetic studies of avermectin biosynthesis in Streptomyces avermitilis.. J Bacteriol..

[36] Ford LM, Eaton TE, Godfrey OW (1990). Selection of Streptomyces ambofaciens mutants that produce large quantities of spiramycin and determination of optimal conditions for spiramycin production.. Appl Environ Microbiol..

